# From Methods to Monographs: Fostering a Culture of Research Quality

**DOI:** 10.1523/ENEURO.0247-23.2023

**Published:** 2023-08-07

**Authors:** Devon C. Crawford, Mariah L. Hoye, Shai D. Silberberg

**Affiliations:** Office of Research Quality, National Institute of Neurological Disorders and Stroke, National Institutes of Health, Bethesda, MD 20892

“Al estudiar las monografías de la especialidad que se desee cultivar, debemos fijarnos sobre todo en dos cosas: en los métodos de investigación de que el autor se ha servido en sus pesquisas, y en los problemas que han quedado pendientes de solución.”

[“When studying monographs from the specialty one wishes to cultivate, we should focus on two things above all: on the investigation methods that the author has used in their research, and on the problems that are still pending a solution.”]

(Ramón y Cajal, 1897) [Translation to English by Devon C. Crawford]

## Introduction

Santiago Ramón y Cajal is renowned for his careful observations and highly detailed drawings of cells within the nervous system. His advice, that our primary focus when reading scholarly communications should be on the methods used and the problems yet to be solved (Ramón y Cajal, 1897), feels especially difficult in the current age. Manuscript formatting guidelines and publication conventions often cause investigators to abbreviate their research methods, leading to important information missing on the design, conduct, and analysis of a study. As a result, it is often challenging to evaluate the quality of the “investigation methods that the author has used in their research” in many research documents and thus to deduce the “problems that are still pending a solution.” With the central role that publications play in both communicating science and assessing researchers for career progression and financial support, thorough methodological reporting is necessary for others to evaluate the quality of the research. Although great scientific advances have been made over hundreds of years and continue to be made today, fully transparent reporting could accelerate progress toward fundamental biological knowledge and treatments for disease.

Understanding the factors that may have led to current practices and norms in communicating science could serve as a guide to redirect the culture of science to better emphasize rigorous and transparent research methods, as Ramón y Cajal advised. The National Institute of Neurological Disorders and Stroke (NINDS), a component of the National Institutes of Health (NIH), has been in recent years promoting enhanced attention to rigor and transparency in the scientific ecosystem via focused programs. Herein we provide historical context for the cultural issues targeted by such programs, describe these efforts, and present a vision for a future that incentivizes research quality commensurate with its vital role in scientific progress. Importantly, bolstering partnerships with the scientific community is vital for elevating scientific rigor and transparent reporting within the scientific culture so that, together, we may help improve the practice of scientific research for the benefit of all.

## The Scientific Ecosystem

**Figure 1.**
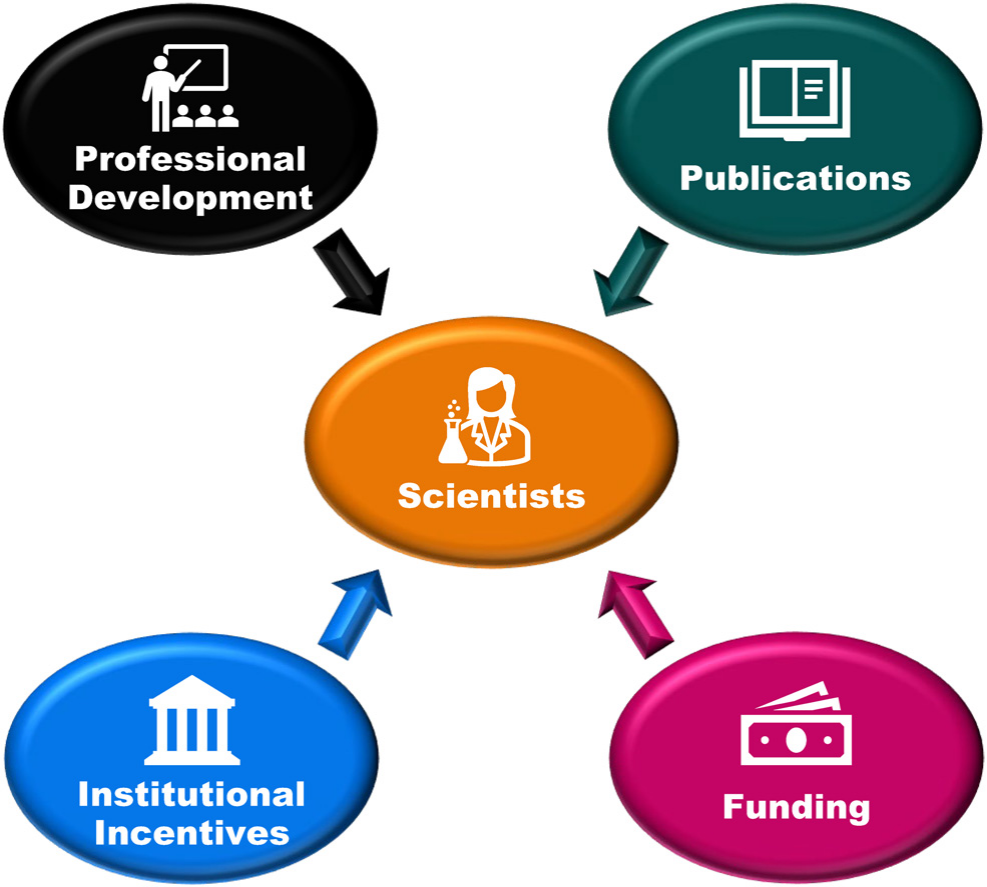
Incentives and pressures in the scientific ecosystem. Although scientists are ultimately responsible for the quality of the research they perform and how it is reported, they are also under numerous pressures from various parts of the scientific ecosystem that can negatively impact rigor and transparency. There are pressures to publish research articles (especially in high-impact journals), obtain funding (which may, in part, depend on publication history), seek professional development opportunities to advance one's career and progress through a career ladder that often uses publication and funding metrics as benchmarks of research performance. This ecosystem tends to incentivize exciting studies that generate hypotheses or incompletely explore hypotheses over carefully designed studies with high research quality and transparency that more deeply explore a scientific question.

There are many contributors to the scientific ecosystem that have, both historically and today, played important roles in defining norms and shaping culture in how science is performed and reported (Fig. 1). Because of the deep-seeded origins of many of today’s practices (emphasized by specific references used throughout this piece), no single person or entity is responsible for creating the current ecosystem nor for changing the direction of the entire culture. Rather, because of their interdependence, each of these entities need to work together for sustained cultural change to be attainable. As stated in 2014 by the at-the-time Director of NIH Francis Collins and Principal Deputy Director of NIH Lawrence Tabak, “We are reaching out broadly to the research community, scientific publishers, universities, industry, professional organizations, patient-advocacy groups and other stakeholders to take the steps necessary to reset the self-corrective process of scientific inquiry” (Collins and Tabak, 2014).

### Publishing norms

Publications have served a vital role in scientific dissemination for hundreds of years (de Solla Price, 1963; Fyfe et al., 2015) and are commonly used by institutions and funders to appraise scientists for career progression and financial support (Mckiernan et al., 2019). Thus, to supply scientists with venues to share their work, both the number of scientific periodicals and the number of articles within those periodicals have increased dramatically over time (de Solla Price, 1975; Bornmann et al., 2021).

Counterintuitively, the rapid expansion of scientific publishing in the last 300 years, including its expansion online, has not necessarily ensured the transparency of reporting important experimental design elements (Menke et al., 2020, 2022). For example, a systematic review of *in vivo* research studies related to neurologic disorders found that most studies did not report how sample sizes were determined or what measures, such as blinding/masking and randomization, were taken to reduce the risk of unconscious biases (Sena et al., 2014). Similar findings were found in an evaluation of preclinical cardiovascular studies (Ramirez et al., 2017) and in an evaluation of papers published by Nature Publishing Group (NPQIP Collaborative group, 2019). Despite the many scientists, publishers, and institutions who place a high value on transparent reporting in manuscripts (Landis et al., 2012; Nature Neuroscience, 2013; McNutt, 2014b; Marcus and whole Cell team, 2016) the widespread nature of this lack of transparency suggests that the culture of not reporting these items regularly will take time and effort to change. Without a detailed account of the experimental methods used in each study, it is not possible to assess the quality of the research, or reliably interpret the results. Insufficiently rigorous or transparent preclinical work can hamper translation to clinical trials, and incomplete evidence regarding experimental treatments can skew interpretation of effectiveness and harms (Turner et al., 2008; Perrin, 2014). This recognition that transparency is vital was expressed centuries ago by William Gilbert, who wrote “How very easy it is to make mistakes and errors [in interpretation] in the absence of trustworthy experiments” (Gilbert, 1600). How can research be assessed, or even built on, if one does not have access to the full experimental design details?

### Researcher assessment

The level of inadequate experimental transparency in the scientific literature raises additional questions about assessment of researchers themselves. Often, the quality and transparency of “investigation methods that the author has used in their research” are not the focus of assessment criteria. Rather, key metrics commonly used by institutions and funders to appraise neuroscientists for hiring, tenure, promotion, and funding include (1) their history of grant funding, (2) the quantity of their publications, (3) the journals in which they publish, (4) the number of times these publications have been cited, and (5) how experts in the field view their work (Vale, 2012; Collins and Tabak, 2014; Begley et al., 2015; Nosek et al., 2015; Moher et al., 2018; Mckiernan et al., 2019). Although expert reviewers should pay particular attention to the rigor and transparency of the science performed, outsized attention is often awarded to the bibliometrics above because of their simplicity. The measurement of journal citation rates was first proposed by P.L.K. Gross and E.M. Gross as a way to assist university libraries in prioritizing highly cited periodicals when making decisions about periodical subscriptions (Gross and Gross, 1927), a practice that was simplified by Eugene Garfield’s popularization of the Journal Impact Factor (JIF), a normalized number of recent citations that a journal has received (Garfield and Sher, 1963; Garfield, 1972). The JIF, originally meant to help libraries identify collections of particular interest to the scientific community, was rapidly adopted as a surrogate measure for the popularity and prestige of a periodical (Schmid, 2017; Larivière and Sugimoto, 2019; Mckiernan et al., 2019). Often conflated with research quality (Mckiernan et al., 2019), such journal prestige does not provide specific information about the rigor of individual experiments nor about an author’s transparency of reporting. Nevertheless, as early as 1976, the JIF was suggested as a tool to “establish publication records for an individual” (Narin, 1976), although the JIF is an average number applied to a highly skewed distribution of publications (Larivière and Sugimoto, 2019). Attributing its value to individual manuscripts is illogical and was warned against by many, include Garfield himself (Garfield, 1963; Seglen, 1997).

These bibliometric norms, which were formed long ago, likely spurred, albeit inadvertently, incentives that are at odds with fully transparent reporting of rigorously conducted research (Collins and Tabak, 2014). As Goodhart’s Law states, “when a measure becomes a target, it ceases to be a good measure” (Hoskin, 1996). If scientists are routinely assessed based on publication metrics, then they naturally would feel pressure to publish frequently in journals with a high JIF. Indeed, scientists have perceived a pressure to publish under this system since at least 1928, when Clarence Case wrote, “The system of promotion used in our universities amounts to the warning, ‘Publish or perish!’” (Case, 1928). It has long been recognized that publishing in these journals provides visibility and prestige to investigators (Rowlands and Nicholas, 2005), and concentrating on submitting manuscripts to a small number of publication venues inevitably increases rejection rates, making such periodicals even more desirable; the more difficult it is to be selected for publication, the more impressive the publication is perceived to be (Reich, 2013). This pressure continues today, as career stability and grant funding, which are both key for academic scientists to stay scientists, rely heavily on evidence of continuous productivity through publication (Rice et al., 2020).

Simultaneously, these norms around JIF have historically disincentivized journals to publish articles that are predicted to receive few citations. Commercial pressures require publishers to compete for visibility in the scientific community as well as subscriptions from institutions and individuals, a pressure that has become even more acute with the rapid expansion of periodicals over time (Falagas and Alexiou, 2008; McGuigan and Russell, 2008). To keep JIF high, journals have often prioritized articles with broad interest in the form of novelty and potential scientific impact (Brumback, 2008; Falagas and Alexiou, 2008). Unexpected and exciting results, however, are often preliminary and need additional, high-quality validation. This pressure to exhibit novelty could promote incomplete studies (such as studies that, despite containing a high volume of experiments and data, do not fully investigate approaches that could refute the hypothesis or do not fully report data inconsistent with the hypothesis), as scientists fear being “scooped” and losing precedence for publishing a particular finding (Reif, 1961). In the words of Collins and Tabak, “Perhaps the most vexed issue is the academic incentive system. It currently over-emphasizes publishing in high-profile journals…[which] encourages rapid submission of research findings to the detriment of careful replication” (Collins and Tabak, 2014). The focus on volume may accidentally incentivize the building of piles of bricks rather than solid edifices, as Bernard Forscher’s 1963 allegory “Chaos in the Brickyard” posits (Forscher, 1963). If carefully explored scientific questions that led to “unexciting” and especially null findings are not prioritized equally by high-profile journals, some scientists may even decide that the most logical decision is to place such results into a “file drawer” indefinitely because of their perceived low value or difficulty in getting them published (Rosenthal, 1979). This last practice, resulting in publication bias, distorts the known body of scientific knowledge and is widespread (Kyzas et al., 2007; Turner et al., 2008; Sena et al., 2010). This environment could also potentially lead to slower career progression and lower success in obtaining funding for some very careful and rigorous scientists.

Despite the value placed on JIF, journal and individual publication citation counts have been shown either not to correlate or to correlate negatively with several dimensions of research quality (Macleod et al., 2015; Dougherty and Horne, 2022). Therefore, many suggest that researcher assessment by institutions and funders should be refocused away from JIF and other bibliometrics (Hicks et al., 2015; Schmid, 2017; Mckiernan et al., 2019; Moher et al., 2020), including Collins and Tabak: “University promotion and tenure committees must resist the temptation to use arbitrary surrogates, such as the number of publications in journals with high impact factors, when evaluating an investigator’s scientific contributions and future potential” (Collins and Tabak, 2014). The same could be said for funding review committees. Assessing research quality may very well require more time and resources to implement, but it would better identify and reward scientists who employ the most rigorous and transparent practices.

### Peer review

Peer review could be regarded as one effective locus for screening publications and grant proposals for research quality. However, this relatively young practice (de Solla Price, 1975; Spier, 2002) is highly dependent on scientific peers subjected to the same messaging that promotes publication productivity and bibliometrics as major foci for assessment. While transparency in reporting one’s methods of experimental design and analysis is vital for enabling peer review of research quality in grant applications and publications, assessing said quality can be challenging. As the number of manuscripts and grant submissions continues to rise, reviewers (who volunteer their time and expertise) are likely asked to evaluate increasing numbers of documents, and thus, for the sake of time, may not be able to pay as deep attention to the “investigation methods that the author has used in their research” as they would like. The scientific complexity within individual publications has also strikingly increased in parallel with their volume (Cordero et al., 2016), possibly because of the pressures described earlier. To illustrate this, we found that the first 18 papers published in *Cell* (January–March 1974) had an average of 3.2 authors, 4.5 figures, and 7.7 panels per paper. In contrast, in the first issue of *Cell* in 2023, comprising 18 papers, these numbers were 19.9, 13.4, and 102.1, respectively. This increased length and complexity of individual publications further adds (1) time to review a single manuscript and (2) in many cases, additional required expertise to evaluate all aspects of a manuscript that individual reviewers may not have.

The expansion of experimental techniques and the growth of team science are exciting developments in the advancement of biomedical research, but they also bring additional responsibilities. Claude Bernard foresaw this contemporary issue as early as 1865: “The more complex the science, the more essential is it, in fact, to establish a good experimental standard, so as to secure comparable facts, free from sources of error” (Bernard, 1865). Although critically important for scientific discourse, the increased time commitment and challenge for peer reviewers, in addition to the competing priorities signaled by institutions and funders, means that peer review alone cannot be expected to solve issues of research quality in scientific proposals and publications. Changes to peer review to enhance attention to rigor and transparency must be part of a broader change in the culture of science.

### Scientific culture

Given the forces that have been at play for many generations of scientists, shifting the focus to more explicitly emphasize research quality through rigor and transparency in scientific communications will require a multipronged approach. Institutions, publishers, funders, professional organizations, and researchers, as contributors to the biomedical landscape, will all need to play their part in this evolution (Fig. 1). Indeed, in recent years there have been several efforts to change the culture of science from many sectors of the research ecosystem, such as proposed changes to funding structures (Liu et al., 2020), publication practices (Chambers, 2013; Berg et al., 2016; Schmid, 2017; du Sert et al., 2020), institutional incentive structures (Dirnagl et al., 2016; Strech et al., 2020), and researcher education (Bosch and Casadevall, 2017; du Sert et al., 2020; Bespalov et al., 2021). To bolster and sustain such efforts, every member of the scientific ecosystem is integral to enhancing research rigor and transparency where possible.

## NINDS Efforts

**Figure 2.**
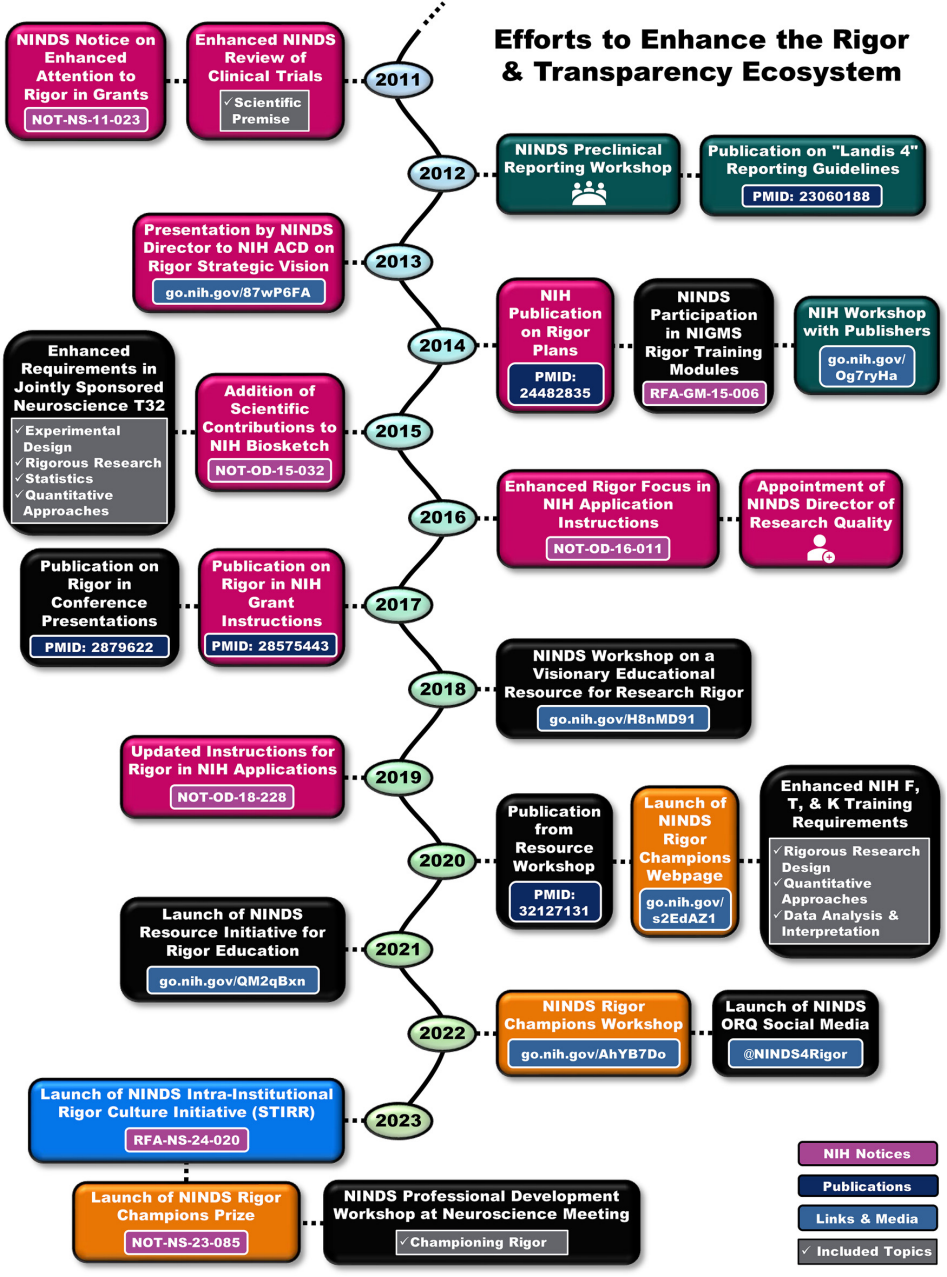
Key milestones in National Institute of Neurological Disorders and Stroke (NINDS) and National Institutes of Health (NIH) efforts to catalyze culture change. Timeline with examples of NINDS and trans-NIH efforts since 2011 to improve the awareness and practice of rigorous and transparent research practices. Corresponding to elements of the scientific ecosystem shown in Figure 1, magenta boxes relate to funding efforts, dark green boxes to reporting in publications, black boxes to training and professional development, orange boxes to efforts of individual rigor champions, and the blue box to institutional culture change. ACD, Advisory Committee to the NIH Director. NIGMS, National Institute of General Medical Sciences. T32, Ruth L. Kirschstein National Research Service Award Institutional Training Program. ORQ, Office of Research Quality.

As a funding entity, NINDS has acknowledged its role in the scientific ecosystem and actively promoted better research practices through efforts to help shift the scientific culture (Fig. 2). As a complement to efforts outside of NINDS, these solutions are designed to disrupt long-ingrained practices and spur action toward improving scientific rigor and transparency to ensure a better future. Many sectors of the scientific ecosystem are targeted by these programs to engage the entire community and catalyze change.

### Funding

Funding is a strong incentive for shaping researcher behavior. NINDS took steps to bolster research quality and transparency of “investigation methods that the author has used in their research” within grant applications by publicly declaring enhanced attention to rigor and transparency in 2011 (https://go.nih.gov/HEQ6Jem) and, around the same time, modifying NINDS-run reviews of clinical trials to pay explicit attention to the research quality of foundational work justifying the trial. This preceded NIH’s modifications to grant application instructions that increased applicant and reviewer focus on the rigor of the prior research (formerly “scientific premise”), rigor of the proposed research, biological variables, and authentication of chemical and biological reagents (https://go.nih.gov/UWck7hx, updated in https://go.nih.gov/8BwyOex). NINDS also created a dedicated Office of Research Quality (ORQ; https://go.nih.gov/kxZASSG), which is unique among the Institutes, Centers, and Offices of NIH, to promote rigorous research practices and transparent reporting within the scientific community. In addition, NINDS bolstered rigor-related language in its funding opportunities (including the review criteria) and strengthened consideration of the investigators’ track record of rigorous research when deciding whether to fund an application beyond the payline (https://go.nih.gov/tffo0yt). NINDS, along with the NIH, continues to transform approaches to funding and peer review with the goal of better identifying and promoting high-quality research.

### Publications

Given that publication practices heavily influence transparency and dissemination of research, NINDS convened a workshop in 2012 with journal editors, funders, peer reviewers, and investigators to identify key practices in preclinical animal studies that should be reported. This led to the recommendation that “at a minimum, studies should report on sample-size estimation, whether and how animals were randomized, whether investigators were blind to the treatment, and the handling of data” (Landis et al., 2012). Some publishers quickly adopted these guidelines (Nature, 2013; Kelner, 2013), and they and NIH subsequently held a workshop with additional editors and publishers that resulted in reporting guidelines that were widely endorsed (McNutt, 2014a). Although transparency in reporting important experimental design practices appears to have increased (NPQIP Collaborative group, 2019), transparency of such items in biomedical research overall could still use improvement (Menke et al., 2020, 2022).

Reporting guidelines, however, are just one way to encourage transparency of “investigation methods.” For example, there are some peer-reviewed journals that focus on rigor and transparency of methods rather than the outcome of experiments (Chambers and Tzavella, 2022), and NINDS is exploring additional ways to address publication bias. There has also been a recent push in the scientific community for more open science beyond traditional publications (Laakso et al., 2011), which includes such efforts as NIH encouraging dissemination of early work through preprints (https://go.nih.gov/b6CETx4) and widely sharing research data regardless of relationship to a publication (https://go.nih.gov/hgKCjqt). Open science, however, does not necessarily guarantee increased reporting of important experimental design elements, nor does it guarantee thorough review by peers. For this reason, it is vital for open science and data sharing efforts to specifically consider reporting of important metadata, such as experimental rigor and transparency practices, to help emphasize research quality alongside access to the resulting data.

### Education and professional development

High levels of rigor and transparency in scientific communications can only be achieved if the community, including early career researchers, are adequately educated about the research quality issues inherent in their experiments as well as the best ways to mitigate them. If researchers do not know there are issues, or they know there are issues but not how to fix them, we cannot expect those issues to change. For this reason, efforts by NINDS to improve research quality have also emphasized the importance of and the need for training and education in the principles of research rigor (Landis et al., 2012; Koroshetz et al., 2020). For example, NINDS drove changes to the Jointly Sponsored Predoctoral Training Program in the Neurosciences (T32), which requires supported students and scholars to obtain a thorough understanding of experimental design, including the principles of experimental rigor (https://go.nih.gov/duZNsQI). Skill-building in such rigorous research design and analysis practices became an NIH-wide requirement for training grants in 2020 (https://go.nih.gov/E7qLJhT).

Despite these training requirements, NINDS noted a dearth of high-quality educational materials and programs devoted to research rigor and transparency among NINDS T32 training grant institutions in 2018 (https://go.nih.gov/6XuP424) and, therefore, held a workshop on how to improve education in the principles of rigorous research (Koroshetz et al., 2020). Following this workshop, NINDS launched an initiative to build an innovative online platform that will host educational units on fundamental principles of research rigor that can be integrated easily into training programs (https://go.nih.gov/L1D5u7N). This initiative is being driven by the scientific community, for the scientific community (https://c4r.io/), and there is one final receipt date remaining to apply for a grant to create additional educational units (https://go.nih.gov/j967x5r). This effort is still early in development, so we encourage the neuroscience community to follow its progress (for example, through ORQ’s social media, see https://twitter.com/NINDS4Rigor) and participate in future meetings or testing of the materials.

Science societies and other professional organizations are also an important locus for professional development. To this end, NINDS often partners with these groups to enhance efforts to educate and provide resources for the scientific community. For example, a 2017 NINDS-hosted roundtable with conference organizers resulted in multiple suggestions for enhanced transparency at conferences, including through adding small icons to talks and posters to signal various rigor and transparency practices with little added researcher effort (Silberberg et al., 2017). ORQ is continuing to pilot the use of such icons at additional NINDS and non-NINDS meetings to gauge interest and effectiveness. Recently, the Society for Neuroscience, which has developed educational materials on rigorous research practices through an NINDS-funded grant (https://neuronline.sfn.org/collection/foundations-of-rigorous-neuroscience-research), has added requirements for annual meeting presenters to provide information on the rigor of their studies (https://www.sfn.org/meetings/meeting-policies-and-guidelines/presenter-guidelines-and-policies-for-sfn-events). This could provide an opportunity for attendees to try out such icons. In addition, NINDS will be organizing a professional development workshop for the upcoming 2023 meeting of the Society for Neuroscience on how to better champion rigorous research practices, which will provide practical examples and advice to individual researchers about how to catalyze change within their own institutions. Education and support from professional organizations can be important catalysts for empowering scientists to shift behavior, initiate new and better practices, and spread the lessons they have learned to others.

### Individual scientists and rigor champions

We encourage motivated individuals who would like to change the culture of science, by elevating the importance of careful research, to act as “rigor champions.” Such individuals with drive and vision can spark broader change than they might realize. They cannot, however, transform the scientific ecosystem alone. Communities for champions to connect with each other are needed for culture change efforts to thrive and be sustained (Orben, 2019; Stewart et al., 2022). Thus, NINDS has created avenues to catalyze these communities to connect and share experiences (https://go.nih.gov/5ZQX6Yv). For example, NINDS hosted a workshop in 2022 to better glean a path for supporting rigor champions (https://go.nih.gov/AhYB7Do), out of which came the clear message that rigor and transparency efforts should be better supported and recognized. To this end, NINDS recently launched the NINDS Rigor Champions Prize (https://www.challenge.gov/?challenge=ninds-rigor-champions-prize), which is a federal Challenge mechanism aiming to recognize individuals and small teams who have worked to enhance culture around scientific rigor and transparency. We especially encourage participation by early career researchers, institutional staff, and other members of the scientific community who do not traditionally apply for NIH grants or receive recognition for their efforts through traditional hiring and promotion criteria. The first round of winners will be announced in November 2023. Through these and similar efforts, we hope to better identify and recognize worthwhile activities already occurring in the community.

### Institutional incentives

For the efforts described above to be successful, career incentives for scientists must align with valuing rigor, transparency, education, and championship of better research practices. Culture at the level of a department or institution can reinforce behaviors and attitudes that enhance or diminish adherence to such practices. Thus, to support positive culture change, NINDS recently released a funding opportunity for United States-based institutional entities that perform or support neuroscience research to create innovative programs, strategies, and approaches that incentivize research quality (https://go.nih.gov/BbnZR29). This incentivization can be achieved through infrastructure, education, adjustments in researcher assessment and recognition, policy change, or a variety of other approaches. We encourage applications that propose programs of various size or focus, so a spectrum of programs is expected. Successful, sustainable programs have the potential to spread to additional institutions, which could catalyze wider change and enhance research quality across the scientific community.

## Looking Forward

Culture change to elevate the rigor and transparency of “investigation methods that the author has used in their research,” per Ramón y Cajal’s advice, can be achieved only with the help of every contributor to the scientific milieu, including scientists, educators, professional organizations, publishers, funders, and institutions (Koroshetz et al., 2020). The incentives and pressures that exist in today’s research environment, which have been deeply rooted for a long time, inevitably affect how scientists interact with the other components in the system. Thus, all must work in concert toward the common goal of improving rigor and transparency for it to be successful. Many groups and individuals have worked to change this culture, and NINDS as a funding entity has a responsibility to contribute positively to a future where the neuroscience community and the biomedical community at large embrace a collective push to solve “problems that are still pending a solution” with the highest quality of work possible. Over time, NINDS’s efforts have built on each other, with new opportunities recently announced to further this collective goal, and additional avenues to address research rigor and transparency issues and to support the scientific community will likely be needed. To emphasize its commitment, NINDS included rigor and transparency as a “cross-cutting strategy” in its 2021–2026 Strategic Plan (https://go.nih.gov/4HyXnv5), promising to “promote scientific rigor and transparency throughout all NINDS programs and policies.” NINDS’s efforts complement those by other entities trying to shift attitudes and practices (such as those by working groups of the Advisory Committee to the NIH Director; see https://go.nih.gov/uhdqIR4), and together these activities will be more effective at initiating and sustaining change. Rigor champions at all levels, fields, and positions can contribute at least a small piece to the larger puzzle, and every piece has value. The collective partnership of everyone in the scientific community is fundamental for a future that values transparent and rigorous research so that knowledge and clinical practice are built on the strongest foundation.

## Synthesis

Reviewing Editor: Christophe Bernard, INSERM & Institut de Neurosciences des Systèmes Decisions are customarily a result of the Reviewing Editor and the peer reviewers coming together and discussing their recommendations until a consensus is reached. When revisions are invited, a fact-based synthesis statement explaining their decision and outlining what is needed to prepare a revision will be listed below. The following reviewer(s) agreed to reveal their identity: NONE.

Please correct some references

I prefer the short legends

And it is always good to see Claude Bernard cited!
